# N6-methyladenine RNA methylation epigenetic modification and diabetic microvascular complications

**DOI:** 10.3389/fendo.2024.1462146

**Published:** 2024-09-04

**Authors:** Yuanyuan Wang, Jiayun Zou, Hua Zhou

**Affiliations:** ^1^ Department of Nephrology, Shengjing Hospital of China Medical University, Shenyang, China; ^2^ Department of Oncology, Shengjing Hospital of China Medical University, Shenyang, China

**Keywords:** N6-methyladensine, writer, eraser, reader, DM, microvascular complications

## Abstract

N6-methyladensine (m^6^A) has been identified as the best-characterized and the most abundant mRNA modification in eukaryotes. It can be dynamically regulated, removed, and recognized by its specific cellular components (respectively called “writers,” “erasers,” “readers”) and have become a hot research field in a variety of biological processes and diseases. Currently, the underlying molecular mechanisms of m^6^A epigenetic modification in diabetes mellitus (DM) and diabetic microvascular complications have not been extensively clarified. In this review, we focus on the effects and possible mechanisms of m^6^A as possible potential biomarkers and therapeutic targets in the treatment of DM and diabetic microvascular complications.

## 1 Introduction

Diabetes mellitus (DM) is an international health problem characterized by insulin resistance (IR) and insulin deficit (1). It has been estimated by the International Diabetes Federation that 537 million individuals worldwide are living with diabetes in 2021. By 2045, 784 million people will be affected by diabetes (2). DM can lead to macrovascular and microvascular complications. Macrovascular complications include coronary heart disease, strokes, and peripheral arterial disease. Microvascular complications include diabetic kidney disease (DKD), diabetic retinopathy (DR), and diabetic peripheral neuropathy (DPN) (3).

Methylation is an important modification of nucleic acids and proteins. It can regulate the expression and inhibition of genes and be involved in a variety of diseases, such as DM, cancer, aging, and so on (1, 4–6). RNA epigenetics modification has become a regulatory mechanism to coordinate cell transcriptome and proteome in different physiological processes. Similar to DNA methylation and histone modifications, RNA modifications can be dynamically regulated, removed, and recognized by its specific cellular components (respectively called “writers,” “eraser,” “readers”) and affect RNA splicing, stability, localization, translation, and transcription of mRNAs (7). RNA methylation includes N6-methyladensine (m^6^A), 5-methylcytosine, N1-methyladenosine, N7-methylguanosine, etc. (8). Among these modifications, m^6^A has been identified as the best-characterized and the most abundant mRNA modification in eukaryotes (9–11). And m^6^A methylation has become a hot research field in a variety of biological processes and diseases, such as aging, lipid metabolism, the development of hematopoietic system, central nervous system and reproductive system, obesity, cardiovascular diseases, cancers, renal diseases and et al. (7, 11–16). We find that five reviews on m^6^A and diabetes have been published (1, 17–20). However, there’re still some gaps that the existing reviews mainly focused on the relationship between m^6^A modification and DM and did not provide a detailed summary about the advancement of m^6^A and diabetic microvascular complications. Microvascular injury is very important for the prognosis of DM. In consequence, this review highlights the molecular mechanisms and potential therapeutic targets of m^6^A and diabetic microvascular complications.

## 2 m^6^A

**Figure 1:**
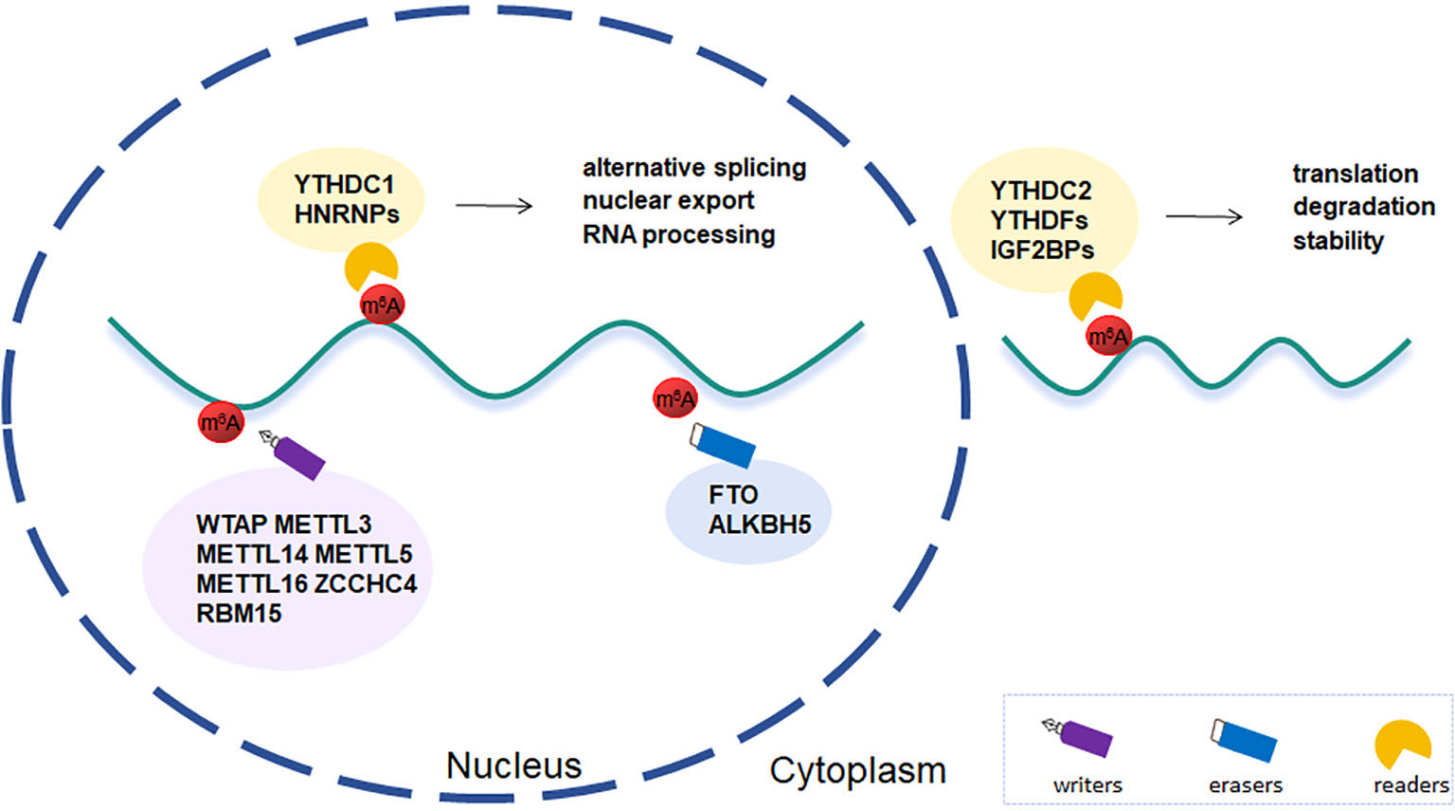
Regulation and function of m^6^A modification.

m^6^A, first discovered in Novikoff hepatic cancer cells (21), is an internal modification and highly clustered in near stop codons and in 3’UTRs of mRNAs (22). With the constant sequence RRACH (where R stands for A or G and H for A, C, or U), it is found in highly conserved sections and is dynamically regulated by particular methyltransferases and demethylases, which interacts to maintain RNA methylation homeostasis (10, 23) (**Figure 1**).

### 2.1 Writers

The m^6^A modification is post-transcriptionally installed by methyltransferase complex(MTC), which composes of METTL3, METTL14, Wilms’ tumor 1-associated protein (WTAP) (24). Either METTL3 or METTL14 alone exhibits fairly weak catalytic activity *in vitro*. However, the METTL3-METTL14 complex displayed significantly higher activity. Meanwhile, METTL14 can offer an RNA-binding scaffold to enhanced activity of METTL3 methylation (25). METTL3 and METTL14 are the core subunits of MTC and play a key role in different biological processes. In mouse embryonic brains, knockout of METTL3 and METTL14 can prolong the cell cycle of radial glial cells and extend cortical neurogenesis into the postnatal stage in a m^6^A-dependent manner (26). Besides, the METTL3-METTL14 heterodimer complex is closely related to the most of m^6^A sites in mRNA. More than 99% of the total m^6^A was lost in mouse embryonic stem cells upon genomic deletion of METTL3 or CRISPR-mediated silencing of METTL14 (27). WTAP is the third subunit of MTC. Although WTAP has no catalytic activity against RNA targets, it can facilitate the accumulation of METTL3-METTL14 heterodimer complex in nuclear speckles (28). WTAP depletion led to a marked decrease of m^6^A levels in mRNA and the buildup of both METTL3 and METTL14 in nuclear speckles (29). Besides, WTAP is involved in regulating transcription and alternative splicing of mRNA (29, 30). Therefore, WTAP, as a regulatory subunit, may play a key role in RNA epigenetic modification.

Apart from MTC, other methyltransferases include METTL5, METTL16, Zinc finger CCHC-type containing 4 (ZCCHC4), RNA-binding motif protein 15 (RBM15), and others. The miCLIP analysis confirmed that METTL5 and ZCCHC4 are highly specific methyltransferases which can respectively install 18S rRNA and 28S rRNA. TRMT112 is indispensable to stability and activation of METTL5 in order to achieve metabolic capacity in cells (31). Recent studies demonstrated the crucial function of METTL5-mediated 18S rRNA m^6^A modification in regulation of tumor development and immune microenvironment (32, 33). It has been identified METTL16 is a conserved eukaryotic methyltransferase. MAT2A transcript encoding SAM synthetase and U6 snRNA are the two methylation targets. METTL16 can bind to mRNA MAT2A 3’UTR hairpins, thereby affecting the splicing and stability of MAT2A pre-mRNA and regulating SAM homeostasis (34). Another methyltransferase, RBM15, belongs to the split ends protein family. The long non-coding RNA X-inactive specific transcript (XIST)-mediated gene silencing requires RBM15 and its paralogue-mediate dadenosine methylation. On the contrary, knockdown of both RBM15 and RBM15b blocked XIST-mediated gene silencing (35). So RBM15 is essential for XIST-mediated X chromosome inactivation. In addition, by interacting with intron-binding splicing factor, SF3B1, RBM15 regulates alternative splicing and megakaryocyte differentiation (36).

### 2.2 Erasers

m^6^A demethylases–the fat mass and obesity-associated protein (FTO) and AlkB homolog 5 (ALKBH5), can directly reverse adenosine methylation, so called erasers. Both belong to the AlkB family dioxygenases (37, 38). FTO distributes in the nucleus and cytoplasm and has different substrates. FTO can mediate nuclear m^6^A and cytoplasmic m^6^Am and m^6^A in mRNA, m^6^A in U6RNA, m^6^Am in snRNA and m^1^A in tRNA (39). Early genome-wide related studies have demonstrated the impact of FTO on human obesity and homeostasis (40, 41). Overexpression of FTO in mice led to increased food consumption and obesity whereas inactivation of FTO resulted in significant weight loss and growth retardation (41, 42). Recent studies have shown that FTO is involved in the occurrence and development of various biological processes, such as neuropsychiatric disorders and tumorigenesis and development, etc. (43, 44). Similar to METTL3, ALKBH5 collocates with nuclear speckles and affects mRNA processing, and eventually has an impact on mRNA export and RNA metabolism. ALKBH5 is highly specific for the demethylation of m^6^A mRNA and no other substrates have been found (38).

### 2.3 Readers

The reader’s ability to recognize is a determining factor in how the m^6^A modification affects targeted RNA metabolism. Currently, the most well characterized readers in eukaryotes include YT521-B homology (YTH) domain family, heterogeneous nuclear ribonucleoproteins (HNRNPs), and insulin-like growth factor 2 mRNA-binding proteins (IGF2BPs). These readers alter RNA function by attaching either directly or indirectly to the m^6^A motifs (45). YTH domain family consists of YTH domain family protein 1-3 (YTHDF1-3) and YTH domain containing protein 1-2 (YTHDC1-2). They process similar and highly conserved YTH domain structure, which is mainly composed of four α-helices and four β-strands (46, 47). YTHDC1 is the only member of the YTH family localizing in the nucleus. The N- or C-terminal sequence of YTHDC1 interacts with hypo- or de-phosphorylated RS domain of SRSF3, which mediates alternative splicing and mRNA export (48–50). YTHDC2 binds U-rich motifs in 3’UTRs of RNAs using a DExD box helicase domain and interacts with the 5’ to 3’ exoribonuclease XRN1, thus promoting translation and degradation of mRNAs (51, 52). There are different views on the effect of YTHDF proteins on m^6^A mRNA. Prevailing canonical model think YTHDF1 enhances mRNA translation, YTHDF2 promotes mRNA degradation, and YTHDF3 has both functions (53–55). A unified model proposed by Zaccara et al. demonstrates that YTHDF proteins are closely related to the degradation of m^6^A-RNAs but not the translation (56). Besides, some evidence pointed the function of YTHDF proteins depends on the context in which they are located (57). YTHDF1 can interact with argonaute 2 and contribute to P-body (a membrane-free organelle involved on post-transcriptional regulation of mRNAs) formation, finally promoting the degradation of the target mRNAs (58). The interaction of YTHDF3 with eukaryotic translation initiation factor 2 alpha kinase 2 facilitates translational processes in oxaliplatin-resistant colorectal cancer (59). HNRNPs include HNRNPAB, HNRNPC, HNRNPG, the most abundant protein of which is HNRNPAB. HNRNPs do not directly bind to m^6^A, but through a mechanism called “m^6^A switch”. That is, m^6^A-dependent RNA structural remodeling can regulates RNA-HNRNPs interactions, thus influencing nuclear events such as gene expression, maturation and processing (60–62). Each of HNRNPs processes high- or low-affinity nucleic acid binding sites that can bind a variety of RNA and DNA sequences (63). For instance, HNRNP A2/B1 contains two RNA recognition motif (RRM), RRM1 and RRM2, which can respectively recognize the AGGG motif and UAG motif (60). Besides, it can regulate alternative splicing and promote processing of pri-miRNAs via interacting with DGCR8 protein in a METTL3-dependent manner (64). HNRNPC is involved in the regulation of premRNA splicing and is crucial for the development of tumors (65, 66). HNRNPG contains the extensive low-complexity regions, N-terminal ~300 amino acids and Cterminal ~58 amino acids (62). HNRNPG can bind a purine-rich motif and indirectly recognize the N6-methyl group through a low-complexity region. Besides, Using an RGG region in the low-complexity region, HNRNPG regulates alternative splicing by interacting with phosphorylated C-terminal domain of RNA polymerase II and m^6^A-modified nascent pre-mRNA (67). IGF2BPs and YTHDF2 impose an opposite role in m^6^A function. YTHDF2 contributes to RNA degradation (53, 56). whereas IGF2BPs can regulate stability and translation of target RNAs (68). Besides, they recognize different targets and share only a small number of binding sites (68).

Chemical labeling and sequencing of m^6^A is crucial for studying the function of m^6^A. The chemical inertness of m^6^A makes it difficult to label directly. The most commonly used high-throughput sequencing technique is methylated RNA immunoprecipitation sequencing (MeRIP-Seq) depending on m^6^A antibody, which only provides 100-200 nucleotide resolution. Based on MeRIP-seq, several strategies, including miCLIP, PA-m^6^A-seq and tMeRIP-seq, improve resolution but cannot quantify m^6^A. And there are some antibody-independent strategies which have the advantage of single-base resolution, such as MAZTER-seq and m^6^A-REF-seq. In addition, several novel chemical labeling methods for m^6^A have emerged. m^6^A-SEAL, a FTO-assisted m^6^A selective chemical labelling method, can specifically enrich m^6^A, but cannot achieve single-base resolution and quantify m^6^A. Compared to m^6^A-SEAL, NOseq and m^6^A-label-seq typify single-base resolution feature. On the downside, NOseq Lacks of specificity and sensitivity and cannot distinguish m^6^A and m^6^Am, while m^6^A-label-seq can only be applied to cellular systems and requires the metabolism of Se-allyl-L-selenohomocysteine (69). Current chemical labelling strategies still have much room for improvement. It is essential and urgent to develop a strategy to achieve single-base resolution and specifically enrich m^6^A independent of antibodies.

## 3 m^6^A and DM

Epigenetics of β-cell include DNA methylation, histone modification, chromatin remodeling and accessibility, mRNA and non-coding RNAs (ncRNAs) modification, etc. (70). It can impact β-cell function and adaptation, and be involved in regulating glycometabolism and insulin secretion (71). m^6^A is the most studied RNA modification and closely related to regulation of islet β-cell function and the progression of DM. Studies have shown that m^6^A content in RNA was differentially expressed in different tissues. It was reduced in the peripheral blood of type 2 diabetes (T2D) patients compared with healthy controls (72, 73) and elevated in the livers of high fat diet (HFD) mice (74, 75).

### 3.1 The writers in DM

mRNA m^6^A methylation plays a major role in the pathogenesis of T2D. METTL3 and METTL14 protein levels were downregulated in whole islets from patients with T2D (76, 77). Knowdown of METTL3 and METTL14 in EndoC-βH1 cells inhibited the insulin/IGF1–AKT–PDX1 signaling and led to the cell cycle arrest and impaired insulin secretion in β-cells (76). Methylglyoxal (MG), as a precursor of advanced glycation end products, is significantly increased in patients with newly diagnosed T2D (78). MG-induced downregulation of METTL3 expression promoted decrease in m^6^A levels in β cells. Besides, METTTL3 plays a protected effect on insulin secretion of β-cell with the evidence that silencing of METTL3 significantly reduced glucose-stimulated insulin secretion (GSIS) through regulating musculoaponeurotic fibrosarcoma oncogene family A (MafA), whereas this process could be reversed by upregulation of METTL3 (79). Li et al. reported similar results that islet β-cell-specific deletion of METTL3 induced β-cell failure, decreased insulin secretion and hyperglycemia (80). Meanwhile, in Pdx1+ pancreatic progenitor cells, absence of METTL3 could inhibit Hdac1 expression and further activate wnt/β-catenin and Notch/Hes1 pathways, leading to hyperglycemia and hypoinsulinemia, along with an atrophic pancreas, reduced islet mass, and abnormal increase in ductal formation (81). Remarkably, METTL3 levels increase significantly in β-cells at the onset of type 1 diabetes but quickly decrease with disease progression. METTL3 silencing enhanced the level of 2′-5′-oligoadenylate synthetase (OAS, an innate immune mediators) by increasing its mRNA stability. Hence m^6^A methylation regulates the OAS innate immune response as a β-cell protective mechanism. In β-cell METTL14 knockout mouse lines, glucose intolerance, decreased insulin secretion and lower body weight could be observed (82, 83). RNA sequencing showed METTL14 deficiency led to the upregulation of genes related to β cell death and inflammatory response (82). The loss of METTL3 and METTL14 suppressed the expression of critical β cell transcription factors Pdx1, MafA, and Nkx6.1 as well as mature β-cell markers Ucn3 and GLUT2 (77). These studies indicate METTL3 and METTL14 are essential for maintaining β cell function and maturation. WTAP, another m^6^A writer, has a similar effect on modulating β cell function. WTAP was downregulated in islet β cells of T2D patients due to lipotoxicity and chronic inflammation. WTAP-betaKO mice displayed severe glucose intolerance and reduction in pancreatic insulin content. So WTAP deletion leads to β cell failure and diabetes (84).

METTL3 is also a key factor in regulating IR. In the liver tissues from patients with T2D and HFD mice, the level of m^6^A and METTL3 was consistently elevated (74, 75). FASN is a metabolism-related protein and its m^6^A modification is involved with the development of IR and T2D (85). METTL3 deletion in HepG2 cells and primary hepatocytes dramatically reduced the phosphorylation of IRb, AKT, and GSK3b and the expression of FASN, thereby improving glucose homeostasis and insulin sensitivity (74). Another study showed METTL3 overexpression brought about liver metabolic disorders and IR. On the contrary, METTL3 ablation plays a protective role through increasing the stability of key genes involved in hepatic lipid and glucose metabolism (75). IR is also one of the key immunopathogenesis of nonalcoholic fatty liver disease (NAFLD) (86). Li et al. used a NAFLD model to investigate the biological function of METTL3-mediated m6A methylation in IR. The overexpression of METTL3-mediated CYP2B6 suppressed phosphorylation of the insulin receptor substrate, finally leading to hepatic IR (87).

### 3.2 The erasers in DM

RNA sequencing showed significant associations of variants in FTO and T2D and diabetic nephropathy (DN) (88, 89). Setum FTO level was significantly downregulated in T2D patients and negatively correlated with m^6^A levels (72, 73, 90). However, The expression of FTO in islets and the interaction between FTO expression and insulin secretion is controversial. Taneera et al. found FTO expression was lower in T2D islets than in non-diabetic islets from cadaver donors. And in glucose-responsive insulin-secreting C-peptide modified human proinsulin (GRINCH) cells and INS-1 cells, silencing of FTO expression led to a reduction in insulin secretion (91, 92). Mechanistically, FTO silencing led to a significant decrease in β-cell functional genes, which compromises pancreatic β-cell function. Meanwhile, the dysregulation of FTO expression leads to impaired mitochondrial function and reduced ATP production, possibly contributing to the pathogenesis of T2D (92). The findings of Fan were at odds with those of Taneera et al., observing that the expression of FTO was high in mouse MIN6 cells. And FTO overexpression significantly inhibited insulin secretion and targeted activating NF-κB pathway via reactive oxygen species (ROS) generation, whereas FTO silence had no effect on insulin secretion (93). This difference may be due to different approach of FTO expression (FTO silence by siRNA, vs. overexpression by lentivirus) and different derivation of *in vitro* models (GRINCH cells were obtained from a clonal rat, while MIN6 cells were derived from murine). Wu et al. revealed autophagy overload could trigger β cell apoptosis and decrease insulin secretion. In glucolipotoxic stress conditions, enhanced-NR3C1 significantly upregulated FTO expression in β-cells and further diminished m^6^A modifications on autophagy related genes(Atg12, Atg5, Atg9a, Atg16l2), which induced hyperactive autophagy and β-cell failure (94). And it is observed that (–)-epigallocatechin 3-gallate, the most predominately active catechin in green tea, promoted FTO degradation and prevented the NR3C1 enhancement-induced oxidative stress, thereby exerting a protective effect on glucose tolerance and β-cell function in β-cell-specific NR3C1-overexpressing mice (95). Therefore, targeting FTO provides new insights into the treatment of diabetes.

Although FTO gene has been implicated in the regulation of β-cell function and insulin secretion, the precise mechanism not fully clarified yet. Additional investigations are required to comprehend the regulation of FTO expression and its potential interactions with other transcription factors influencing β-cell survival, metabolism, and function.

### 3.3 The readers in DM

It has been demonstrated that m^6^A reader proteins are crucial in regulating β cell activity and glucose metabolism. In pancreatic β cells from T2D patients, Li et al. found a substantial drop in YTHDC1, which is linked to lipotoxicity and chronic inflammation. In β-cell specific YTHDC1 knockout mice, GSIS was reduced and serum glucagon levels were increased dramatically (96). Similarly, another study showed the expression of m^6^A and YTHDC1 was downregulated in white blood cells from T2D patients. Ablation of YTHDC1 in β-cells of adult mice exhibits a significant decrease in insulin synthesis and secretion, as well as glucose intolerance. On a molecular level, multiple genes correlated with β-cell maturity, such as MafA, Gck and Glut2, were decreased, indicating that β-cell maturity is impacted by YTHDC1 loss (97).

**Figure 2:**
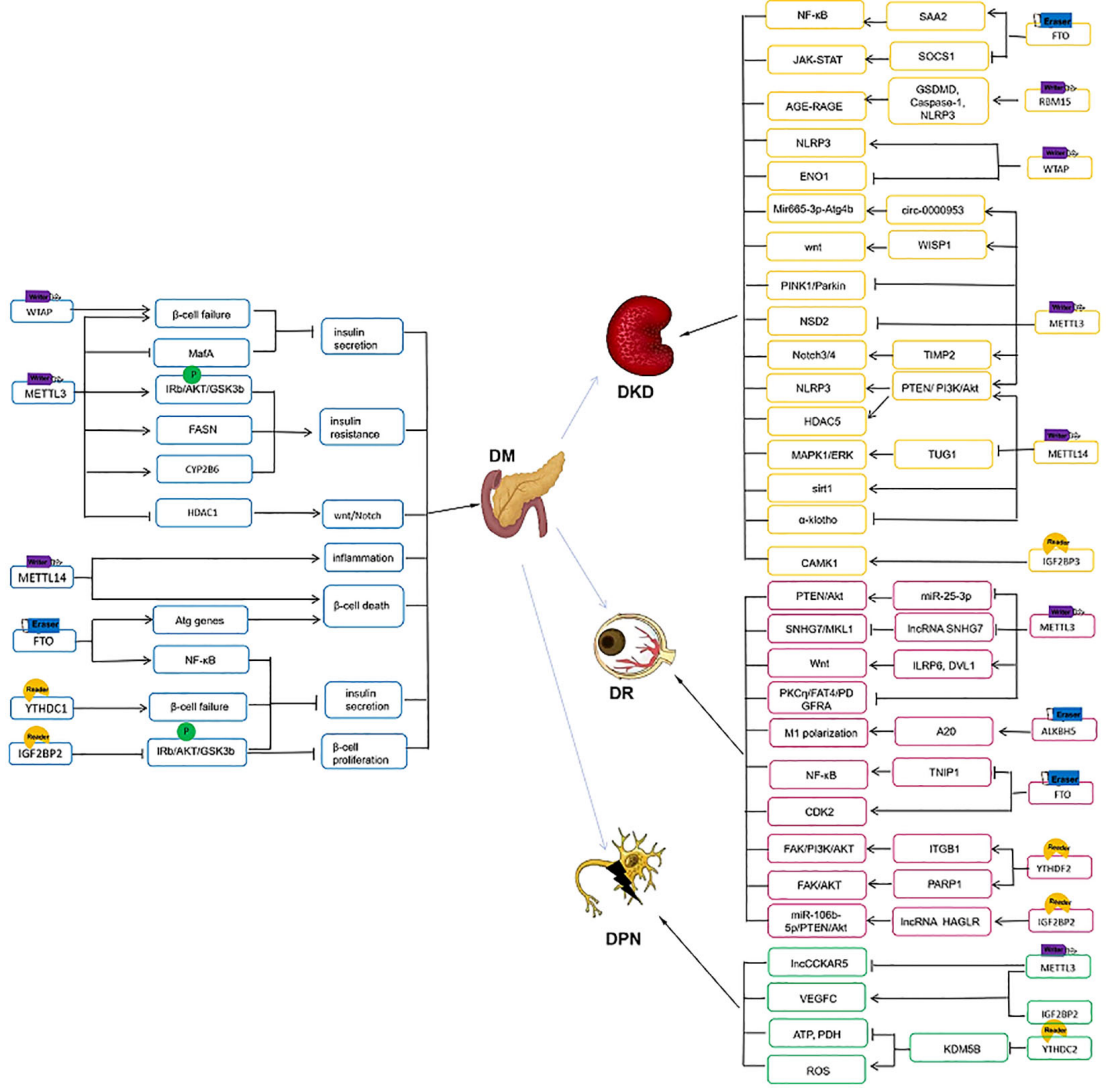
The m^6^A methylation in DM and its microvascular complications.

A cluster of single nucleotide polymorphisms in the second intron of IGF2BP2 found by genome-wide association studies (GWAS) are the susceptibility gene regions of T2D and closely associated with development of T2D/glucose metabolism (98–102). As demonstrated by Regué et al. IGF2BP2 is strongly expressed in pancreatic β cells, which stimulates insulin production through the upregulation of the AKT-GSK3b-PDX1 pathway (103). PDX1 is a critical transcription regulator for the development and maturation of β cell (104). IMP2 deficiency led to a decrease in Pdx1, in turn affecting β-cell proliferation and function (103). Taken together, IGF2BP2 is a human T2D-associated gene. Targeting IGF2BP2 is a promising avenue to improve β-cell function and the development of T2D (**Figure 2**).

ncRNAs are crucial regulatory RNA, including microRNAs (miRNAs), long noncoding RNAs (lncRNAs) and circular RNAs (circRNAs) and exert a potential role in the occurrence and development of DM and its complications (105, 106). A variety of ncRNAs regulate pancreatic β cell survival and insulin secretion (105). There are few studies associated to the effect of m^6^A on ncRNAs in diabetes. A study showed LncRNA XIST was upregulated in the peripheral blood of gestational DM patients and HG-cultured HTR8/SVneo cells, and METTL14 facilitated proliferation and migration and inhibited cell apoptosis and cell cycle arrest by impeding XIST expression (107).

Taken together, these data indicate that m^6^A and its downstream pathways are important regulatory mechanisms in the occurrence and development of diabetes. There are still unresolved issues in this field. In the current investigations, hyperglycemia and hyperlipemia are the most widely used stimulation conditions in experimental model. The majority of research focus on how one enzyme contributes to the pathophysiology of DM. However, DM is a complex and heterogeneous disorder that can be caused by several different factors, such as autoimmune, genetics, environment, lifestyle, etc. (108). There may exist differences in m^6^A modification network under the single condition and the complex pathogenesis of DM. Exploring the pathogenesis of m^6^A in DM under different backgrounds is conducive to elucidate the pathophysiological mechanisms of different diabetes subtypes, so as to provide precise and individualized management strategies for patients in the future.

## 4 m^6^A and microvascular complications of DM

### 4.1 m^6^A and DKD

DKD is associated with an immune cell-mediated inflammatory response. One study has proved that m^6^A-modified lncRNA could mediated the expression and inflammatory response of macrophages in patients with DN (109). DN has multiple morphological changes, including thickened glomerular basement membrane, mesangial expansion, podocyte injury, tubulointerstitial fibrosis, epithelial-to-mesenchymal transition (EMT), etc. (110). EMT is thought to be a key factor in renal fibrosis (111). Some researchers have confirmed m^6^A epigenetics has an important impact on the development of DN through a variety of mechanisms.

#### 4.1.1 The writers in DKD

METTL3/METTL4/WTAP complex, as writers of m^6^A, acted as a regulator in the pathogenesis of DKD. METTL14 expression and m^6^A RNA levels were upregulated in DKD model. METTL14 could promote cell apoptosis and inflammation and aggravated renal injury of DN through three mechanisms (112–114). First, Overexpression of METTL14 increased inflammatory factors levels and apoptosis in human renal glomerular endothelial cells via downregulating m^6^A modification of α−klotho (112). Second, METTL14-mediated RNA m^6^A modification inhibited autophagy and increased apoptosis and inflammation in podocytes and db/db mice through promoting Sirt1 mRNA m^6^A modification and degradation (113). Third, endoplasmic reticulum stress (ERS) can lead to cell apoptosis and is a vital pathogenic mediator of DN (115). METTL14 regulated the m^6^A modification of TUG1 and activated the MAPK1/ERK signaling, which aggravated high glucose (HG)-induced renal tubular epithelial cell apoptosis and ERS (114). According to another study, FTO, METTL3 and METTL14 mRNA were shown to be considerably lower in HK2 cells treated with HG as opposed to normal glucose, whereas only METTL14 overexpression could inhibit the expression of EMT-related proteins, such as TGF-β1 and α-SMA, as well as HDAC5 by regulating Akt pathway (116). Similar to METTL14, METTL3 were also involved in the pathogenesis of DN through several pathways. First, upregulation of METTL3 promoted podocytes apoptosis and inflammation factors levels. And TIMP2-mediated Notch signaling pathway was the downstream target of METTL3 in DN (117). Second, nuclear receptor-binding SET domain protein 2 (NSD2), a SET histone methyltransferase family member, was down-regulated in T2D and promoted the proliferation of pancreatic β cell lines and the release of insulin (118). In DN, METTL3 promotes NSD2 expression to lessen mesangial cell activation and interstitial fibrosis under the HG treatment (119). Third, METTL3 silencing could suppress the proliferation, EMT, migration, and fibrosis of HG-treated HK2 cells through mediate m^6^A modification of WISP1 mRNA, thus alleviating renal injury of DN (120). Fourth, METTL3 could induce apoptosis and mitophagy of renal tubular epithelial cells through modulating the PINK1/Parkin signaling pathway in an YTHDF2-dependent manner, whereas METTL3 knockdown inhibited the progression of DKD (121). Besides, Liu et al. has showed a renoprotective effect of the total flavones of Abelmoschus manihot (TFA) on DN. Mechanistically, TFA could ameliorate pyroptosis and podocytes injury in HG circumstances by downregulating METTL3-dependent m^6^A modification and activating NLRP3-inflammasome and PTEN/PI3K/Akt signaling (122). Another study revealed that silencing of METTL3 suppressed the degradation of circ-0000953 in HG-stimulated podocytes. And the overexpression of circ-0,000,953 ameliorated podocyte injury and autophagy disorder by targeting Mir665-3p-Atg4b (123). It has been proved WTAP is highly expressed in DN patients and in HG−induced HK−2 cells. WTAP promoted cell pyroptosis and inflammation by targeting NLRP3 in DN. Otherwise, WTAP silencing could inhibit DN progression (124). Bai et al. confirmed marrow mesenchymal stem cells (MSCs) administration could alleviate HG-induced HK-2 cells injury and renal injury in DN mice. Mechanistically, MSCs could repress WTAP expression via inactivating Smad2/3 and thus alleviate the development of DN (125). So targeting m^6^A through the writer is a prospective therapy strategy for DN.

RBM15 is also a member of the m^6^A methyltransferases. Qin et al. proved the expression of METTL16 and RBM15 was elevated in the model group of DN mice. In HG-induced HK-2 cells, cell viability was suppressed and the expression of inflammatory factors and pyroptosis-related proteins were elevated, which could be reversed by RBM15 silence. AGE-RAGE signaling pathway activated by RBM15 participated in the pathogenesis of DN (126).

#### 4.1.2 The erasers in DKD

The biological role of FTO-mediated m^6^A modification in DKD are controversary. serum FTO level was decreased in DKD patients (72, 90), whereas the expression of FTO was increased in high glucose-induced podocytes, and FTO upregulation enhanced serum amyloid A2 mRNA stability by regulating the NF-κB pathway, thus participating in podocyte injury and the progression of DKD (127). Another study showed FTO have a protective effect on DKD pathogenesis with evidence that FTO overexpression significantly attenuates kidney injuries and inflammation of DKD via inhibiting SOCS1/JAK/STAT axis (90).

#### 4.1.3 The readers in DKD

Podocyte is an important component of GBM. Podocyte loss and foot process effacement contribute to the development of DKD (128). Insulin-like growth factor-2 is identified to be produced by the glomerular podocyte and is important for maintaining podocyte survival and glomerular function (129). Previous study showed that calcium/calmodulin-dependent protein kinases (CAMK), belongs to CAMKs Ser/Thr protein kinase family, play an important role in maintaining mitochondrial homeostasis and regulating inflammation and oxidative response (130, 131). IGF2BP3 promoted the stability of CAMK1 mRNA by m^6^A modification and further alleviated DN progression via inhibiting mitochondria fission and cell apoptosis (132). Lin et al. found circUBXN7 was significantly upregulated in DKD plasma. And upregulated circUBXN7 enhanced the binding of IGF2BP2 and SP1 mRNA, which promoted macrophage infiltration, tubular EMT and fibrosis and accelerated the progression of DKD (133) (**Figure 2**, **Table 1**).

**Table 1:** The regulatory mechanism of m^6^A in DKD.

Patients/Animal/Cell model		m^6^A Effector Protein	Target genes	Mechanisms	Author and Year of Publication
patients sample	DN patients renal biopsy samples	METTL14 ↑	α-klotho ↓	cell/renal injury, inflammation and apoptosis↑	Li et al., 2021 (112)
animal model	db/db mice kidney				
cell model	HG induced HRGECs				
patients sample	DN patients renal biopsy samples	METTL14 ↑	sirt1 ↑	apoptosis and inflammation ↑	Lu et al., 2021 (113)
animal model	db/db mice kidney				
cell model	human podocytes				
animal model	STZ-induced mice kidney	METTL14 ↑	TUG1↓	MAPK1/ERK signaling ↑ — cell apoptosis and ERS/renal lesions and fibrosis ↑	Zheng et al., 2023 (114)
cell model	HG-induced HK2 cell				
animal model	STZ-induced mice kidney	METTL14 ↓	PTEN ↓	PI3K/Akt pathway↑ —HDAC5↑ —EMT ↑	Xu et al., 2021 (116)
cell model	HG-induced HK2 cell				
patients sample	DN patients renal biopsy samples	METTL3 ↑	TIMP2 ↑	Notch3 and Notch4 signaling↑ — podocyte injury, apoptosis and inflammation ↑	Jiang et al., 2022 (117)
animal model	db/db mice kidney STZ-induced mice kidney				
cell model	HG-induced MPC5 cell				
patients sample	DN patients renal biopsy samples	METTL3 ↓	NSD2 ↓	mesangial cell activation and interstitial fibrosis ↑	Tang et al., 2022 (118)
animal model	STZ-induced mice kidney				
cell model	mouse mesangial cell line				
animal model	STZ-induced mice kidney	METTL3 ↑	WISP1 ↑	Wnt/β-catenin pathway ↑ —proliferation, EMT, migration, and fibrosis ↑	Chen et al., 2024 (120)
cell model	HG-induced HK2 cells				
animal model	STZ-induced mice kidney	METTL3 ↑	PINK1/Parkin ↓	apoptosis and mitophagy ↑	Wang et al., 2023 (121)
cell model	HG-induced HK2 cells				
cell model	HG-induced MPC5 cell	METTL3 ↓	PTEN ↑	PI3K/Akt Signaling ↓— NLRP3↑—pyroptosis and cell injury ↑	Liu et al., 2021 (122)
patients sample	renal biopsy samples of DN patients	METTL3 ↓	circ-0000953 ↑	Mir665-3p-Atg4b ↑— podocyte injury and autophagy ↓	Liu et al., 2024 (123)
animal model	STZ-induced mice kidney db/db mice kidney HFD mice kidney				
cell model	HG-induced podocytes				
patients sample	renal biopsy samples of DN patients	WTAP ↑	NLRP3 ↑	cell pyroptosis and inflammation ↑	Lan et al., 2022 (124)
animal model	db/db mice kidney				
cell model	HG-induced HK2 cells				
animal model	STZ-induced mice kidney	WTAP ↓	ENO1 ↓	renal injury and inflammation ↓	Bai et al., 2024 (125)
cell model	HG-induced HK2 cells				
animal model	db/db mice kidney	RBM15 ↑	GSDMD, Caspase-1, NLRP3 ↑	AGE-RAGE signaling ↑—cell pyroptosis and inflammation ↑	Qin et al., 2023 (126)
cell model	HG-induced HK-2 cells				
animal model	DN patients renal biopsy samples	FTO ↓	SOCS1 ↓	JAK-STAT pathway ↑ —kidney inflammation and injury ↑	Sun et al., 2022 (90)
cell model	db/db mice kidney				
animal model	STZ-induced mice kidney	FTO ↑	SAA2 ↑	NF-κB pathway ↑—podocyte injury and inflammation ↑	Lang et al., 2024 (127)
cell model	human podocytes				
animal model	STZ-induced mice kidney	IGF2BP3 ↑	CAMK1 ↑	mitochondria fission and cell apoptosis ↓	Yuan et al., 2024 (132)
cell model	HG-induced HK2 cell				

The up arrow (↑) means increased and the down arrow (↓) means decreased. METTL14, methyltransferase-like 14; METTL3, methyltransferase-like3; WTAP, Wilms’ tumor 1-associated protein; RBM15, RNA-binding motif protein 15; FTO, fat mass and obesity-associated protein; IGF2BP3, insulin-like growth factor 2 mRNA-binding protein 3; DKD, diabetic kidney disease; DN, diabetic nephropathy; HG, high glucose; HRGECs, human renal glomerular endothelial cells; STZ, streptozotocin; HK2, human renal tubular epithelial cells; MPC5, mouse podocyte cell-5 line; TUG1, taurine upregulated gene 1; ERS, endoplasmic reticulum stress; HDAC5, histone deacetylase 5; EMT, epithelial–mesenchymal transition; NSD2, nuclear receptor-binding SET domain protein 2; PINK1, PTEN induced putative kinase 1; PTEN, phosphate and tension homology; NLRP3, The NOD-like receptor pyrin domain-containing protein 3; MSCs, Marrow mesenchymal stem cells; ENO1, α-enolase; SOCS1, suppressors of cytokine signaling 1; SAA2, serum amyloid A2; CAMK1, calcium/calmodulin-dependent protein kinase type 1.

Current researches have showed that m^6^A methylation is involved in the pathogenesis of DKD through regulating cell injury, inflammation, apoptosis, EMT, interstitial fibrosis and etc., which holds promising implications for its diagnosis and treatment. We found that the same effector protein was differentially expressed in DKD (See **Table 1**) This may be caused by the heterogeneity of different cell or animal models and stimulation conditions, etc. The targeting of m^6^A methylation and effector protein is a promising regulatory mechanism, which will facilitate the advancement of future therapies for DKD, delay the progression of DKD to end-stage renal disease and enhance the overall prognosis of DKD.

### 4.2 m^6^A and DR

DR, a major ocular complication of diabetes, is one of the main causes of visual loss and blindness and accounts for about 30% to 40% of all diabetes cases (134).

#### 4.2.1 The writers in DR

Endothelial dysfunction and EMT are the prominent factors in the pathogenesis of DR (135, 136). Retinal pigment epithelium (RPE) cells are essential for the development and maintenance of adjacent photoreceptors in the vertebrate retina and frequently utilized *in vitro* cellular models in DR research (137). METTL3 expression levels were lowered in RPE cells treated with HG in a time-dependent manner. Mechanistically, overexpression of METTL3 in RPE cells attenuated HG-induced cell proliferation, apoptosis, and pyroptosis by regulating miR-25-3p/PTEN/Akt signal pathway (138). Similarly, another study revealed METTL3 expression was downregulated in DR patients, mice and human retinal microvascular endothelial cells. METTL3 overexpression could suppress EMT-related molecules levels via the SNHG7/MKL1 signaling pathway (139). Therefore, METTL3 play a protective role on endothelial dysfunction and EMT.

Oxidative stress is a key event that contributes to DR pathogenesis (140). Under the hypoxic-stress condition, m^6^A methylation and METTL3 in endothelial cells and mouse retinas were upregulated, which contributed to the progression of pathological angiogenesis by regulating wnt signaling activation (a significant increase in LRP6 and DVL1 levels) in a YTHDF1-dependent manner. Conversely, METTL3 silencing suppresses pathological angiogenesis (141). Adequate pericyte attachment was critical for maintenance of blood-retinal barrier (BRB) integrity and maturation. Pericyte dropout impaired BRB, eventually leading to blindness, which is involved in DR pathogenesis and accelerates DR progression (142). Suo et al. reported that m^6^A modification level and METTL3 were increased in retinal pericyte dysfunction under HG condition and retinal vessels of diabetic mice. Pericyte-specific METTL3 deletion resulted in a high expression of PKC-η, FAT4, and PDGFRA via YTHDF2-dependent pathway, which could minimize pericyte apoptosis via impacting their proliferation, viability, and differentiation, and alleviate retinal vascular leakage (143).

#### 4.2.2 The erasers in DR

Retinal microglia’s M1 polarization was enhanced while M2 polarization was suppressed by HG. A20, anti-inflammatory molecule, was negatively correlated with M1 polarization. Besides, inhibiting ALKBH5 in microglia led to higher m^6^A modification level, which decreased A20 expression and further enhanced M1 polarization of retinal microglia of DR. Therefore, targeting A20 is a promising therapeutic means for DR (144). FTO is regarded as an essential epitranscriptomic regulator in diabetes-induced vascular endothelial dysfunction. Zhou et al. identified high glucose could induce retinal vascular leakage and enhance inflammation cytokine (IL-1β, IL-18) secretion and apoptosis of human retinal microvascular endothelial cells (HRMECs). FTO silencing could alleviate diabetes-related retinal vascular dysfunction and inflammation both *in vivo* and *in vitro* by inhibiting NF-κB pathway (145). Besides, Chen et al. found in neural retinas collected from STZ mice FTO overexpression contributed to DR phenotypes, including angiogenesis, vascular leakage, inflammation and neurodegeneration by enhancing CDK2 mRNA stability in an YTHDF2-dependent manner (146).

#### 4.2.3 The readers in DR

YTHDF2, m^6^A reader, plays a significant role in the progress of DR. Qi et al. reported the expression of YTHDF2 was significantly decreased in the retinal tissues of STZ-induced mice and HG-treated HRMECs and retinal Müller cells (rMCs). YTHDF2 silencing enhanced expression of pro-inflammatory factors in rMCs and induced proliferation, migration and invasion in HRMECs (147). Besides, high glucose promotes poly (ADP-ribose) polymerase (PARP) expression, which participates in HRMECs apoptosis and mediates retinal fibrosis and inflammation. YTHDF2- mediated m^6^A modification epigenetically may regulate stabilization of m^6^A methylated PARP1 transcripts and activate FAK/AKTsignaling pathway in the pathogenesis of DR (148). Previous study showed the activation of PI3K/AKT pathway led to RPE cells damage and was involved to DR progression (149). YTHDF2 promoted instability of integrin β1 mRNA, which further suppressed FAK/PI3K/AKT pathway and alleviated the progression of DR (147). Dysregulation of autophagy and pyroptosis in RPE cells was a significant pathological mechanism of DR (138, 150). It was reported CircFAT1 bound to YTHDF2 to promote autophagy and suppress pyroptosis of HG-induced RPE cells, thereby alleviating DR progression (151) Another study showed IGF2BP2 may positively regulate lncRNA HOXD Cluster Antisense RNA 1 (HAGLR) via a m^6^A‐dependent manner. Knockdown of HAGLR inhibited HG‐induced HRPE cells apoptosis and pyroptosis via targeting miR‐106b‐5p/PTEN/Akt signaling, thereby alleviating DR pathology (152) (**Figure 2**, **Table 2**).

**Table 2:** The regulatory mechanism of m^6^A in DR.

Patients/Animal/Cell model		m^6^A Effector Protein	Target genes	Mechanisms	Author and Year of Publication
patients sample	The peripheral venous blood samples of T2D patients	METTL3 ↓	miR-25-3p ↓	PTEN/Akt signal↑—cell proliferation, apoptosis and pyroptosis ↑	Zha et al., 2020 (138)
cell model	HG-induced human RPE cell line ARPE-19				
patients sample	human vitreous humor samples from patients with DR	METTL3 ↓	lncRNA SNHG7 ↓	MKL1 signaling ↓—EMT ↑	Cao et al., 2022 (139)
animal model	retinal tissues of STZ-induced mice				
cell model	HG-induced human retinal microvascular endothelial cells				
animal model	OIR Model	METTL3 ↑	LRP6, DVL1↑	wnt signaling↑—pathological angiogenesis↑	Yao et al., 2020 (141)
cell model	hypoxic-stress HUVECs				
animal model	retinal tissues of STZ-induced mice	METTL3 ↑	PKC-η/FAT4/PDGFRA ↓	retinal pericyte loss, vascular leakage, and vascular lesions ↑	Suo et al., 2022 (143)
cell model	HG-induced human retinal pericytes				
animal model	retinal microglia of STZ-induced diabetes rats	ALKBH5 ↑	A20 ↑	M1 polarization ↓	Chen et al., 2022 (144)
cell model	HG-induced mouse microglia cell line BV2				
patients sample	retinal fibrovascular membranes of PDR patients	FTO ↑	TNIP1 ↓	NF-κB pathway ↑—retinal vascular dysfunction and inflammation ↑	Zhou et al., 2023 (145)
animal model	retinas of STZ-induced mice				
cell model	HG-induced HRMECs				
patients sample	fibrovascular membranes obtained from PDR patients	FTO ↑	CDK2 ↑	angiogenesis, vascular leakage, inflammation and neurodegeneration ↑	Chen et al., 2024 (146)
animal model	retinas of STZ-induced mice				
cell model	HG-induced HRMECs				
animal model	retinal tissues of STZ-induced diabetic mice	YTHDF2 ↓	ITGB1 ↑	FAK/PI3K/AKT signaling pathway ↑ —inflammation and neovascularization ↑	Qi et al., 2021 (147)
cell model	HG-induced HRMECs and rMCs				
patients sample	human vitreous samples from PDR patients	YTHDF2 ↓	PARP1↑	FAK/AKT signaling pathway ↑ — cell apoptosis, retinal fibrosis, inflammation ↑	Sun et al., 2022 (148)
animal model	vitreous samples from STZ-induced diabetic rats				
cell model	HG-induced HRMECs				
patients sample	The peripheral venous blood from PDR patients	IGF2BP2 ↑	lncRNA HAGLR ↑	miR‐106b‐5p/PTEN/Akt signaling ↑—apoptosis and pyroptosis ↑	Luo et al., 2023 (152)
cell model	HG-induced human RPE cell				

The up arrow (↑) means increased and the down arrow (↓) means decreased. METTL3, methyltransferase-like3; ALKBH5, AlkB homolog 5; YTHDF2: YT521-B homology N6 methyladenosine RNA binding protein 2; FTO, fat mass and obesity-associated protein; DR, diabetic retinopathy; DM, Diabetes mellitus; RPE, retinal pigment epithelium; STZ, streptozotocin; OIR, Oxygen-induced retinopathy; HUVECs, human umbilical vein endothelial cells;PDR, proliferative diabetic retinopathy; HRMECs, Human retinal microvascular endothelial cells; rMCs, retinal Müller cells; SNHG7, small nucleolar RNA host gene 7; MKL1; megakaryocytic leukemia 1; EMT, epithelial–mesenchymal transition; ITGB1, integrin β1; PARP1, Poly (ADP-ribose) polymerase 1; HAGLR, HOXD Cluster Antisense RNA 1.

The above findings reveal m^6^A RNA modification influences various factors associated with early DR pathogenesis like inflammation, oxidative stress, and neurogenesis, suggesting m^6^A may play a crucial role in metabolic memory of DR. Thus far, only a small number of pathways related to the pathogenesis of DR have been identified. Therefore, to ascertain the underlying regulatory mechanisms of m^6^A methylation in DR, more research is necessary.

### 4.3 m^6^A and DPN

DPN is among the most common long-term complications of diabetes and is at higher risk of all-cause and cardiovascular mortality. Mild symptoms encompass numbness and tingling. Even in some patients it can cause diabetic foot ulcers (DFU), disabling neuropathic pain and lower-limb amputation (153). DPN was characterized by the increase of oxidative stress, mitochondrial damage, and neuron apoptosis (154). Adipose derived stem cells (ADSCs) play a vital role in wound repair by secreting some natural growth factors (155) and activating the PI3K/Akt signaling pathway (156). Zhou et al. discovered a novel link between ADSCs and wound repair with evidence that ADSCs promoted the expression of vascular endothelial growth factor C and lymphangiogenesis marker, LYVE-1, via METTL3/IGF2BP2-m^6^A pathway in DFU mice (157). Wang et al. ‘s study revealed that knocking down METTL3 substantially reduced the abundance of lncCCKAR5, which further inhibited human umbilical cord mesenchymal stem cells apoptosis and promoted macrophage polarization and revascularization under the conditions of HG stimulation. So m^6^A-modification of lncCCKAR-5 is a potential therapeutic target of diabetic wound healing (158). Another study showed HG-treatment resulted in a decrease in ATP as well as PDH activity and an increase in ROS in RSC96 cells, which were reversed by YTHDC2 overexpression. It means YTHDC2 overexpression improved mitochondrial metabolic reprogramming in DPN (159) (**Figure 2**, **Table 3**).

**Table 3:** The regulatory mechanism of m^6^A in DPN.

Patients/Animal/Cell model		m^6^A Effector Protein	Target genes	Mechanisms	Author and Year of Publication
animal model	DFU mice	METTL3/IGF2BP2↑	VEGFC ↑	migration and tubule formation ability of LECs↑ and lymphangiogenesis in DFU mice ↑	Zhou et al., 2021 (157)
cell model	ADSCs-stimulated LECs				
cell model	HG-treated hUCMSCs	METTL3 ↓	lncCCKAR5 ↓	Inhibited hUCMSCs apoptosis and promoted macrophage polarization and revascularization	Wang et al.,2024 (158)
animal model	sciatic nerves of db/db mice	YTHDC2 ↓	KDM5B ↓	mitochondrial metabolic reprogramming ↓	Jiao et al., 2023 (159)
cell model	HG-induced RSC96 cells				

The up arrow (↑) means increased and the down arrow (↓) means decreased. DPN, diabetic peripheral neuropathy; METTL3, methyltransferase-like3; IGF2BP2, insulin-like growth factor 2 mRNA-binding protein 2; YTHDC2, YTH domain containing protein 2; DFU, diabetic foot ulcer; ADSCs, adipose derived stem cells; LECs, lymphatic endothelial cells; VEGFC, vascular endothelial growth factor C; hUCMSCs, human umbilical cord mesenchymal stem cells; RSC96, rat Schwann cells; KDM5B, lysine demethylase 5B.

Overall, the evidence so far suggests that m^6^A RNA modification process is emerging as a novel mechanism in DPN, but there are still few relevant studies. Therefore, further probing the molecular mechanism of m^6^A in DPN is of great significance for elucidating the pathogenesis and discovering new therapeutic strategies for DPN.

## 5 Clinical implications of m^6^A modification

The exploration of molecular mechanism is for clinical application. Developing therapies that targeting m^6^A modification or related enzymes has been the focus of many research teams and some m^6^A inhibitors have been discovered (160, 161). Several inhibitors targeting m^6^A have been reported in metabolic diseases. Dac51, entacaponea and meclofenamic acid are the inhibitors of FTO. Dac51 could protect against excessive autophagy activation and reverse β-cell dysfunction (94). Entacapone could decrease fasting blood glucose and improve glucose tolerance in high-fat diet-induced obese mouse model (162). Meclofenamic acid has been shown to alleviate ROS accumulation and cell apoptosis (163). METTL3-specific inhibitor STM2457 has a significant inhibitory effect on renal fibrosis (164). In addition, some natural compounds have shown potential therapeutic effects via targeting m^6^A methylation. Epigallocatechin gallate is the most biologically active and abundant catechin in green tea and curcumin is a natural phenolic compound, which both act to inhibit lipogenesis. Mechanistically, epigallocatechin gallate can suppress the protein stability of FTO while curcumin can decrease the expression of ALKHB5 (95, 165, 166). Another study reported that intake of betaine inhibits hepatic fat accumulation and regulates mitochondrial activity by targeting FTO, thereby improving fatty liver disease and metabolic syndrome (167). TFA, a compound that is extracted from abelmoschus manihot, has been identified to ameliorate pyroptosis and podocytes injury in DKD by targeting METTL3-dependent m^6^A modification (122). The inhibition of dysregulated m^6^A effector proteins is a possible new treatment approach, but no m^6^A inhibitors have entered clinical trials to yet. Researchers still need to devote more efforts to finding new methods and drugs that can be put into clinical use as soon as possible.

Although current studies reveals the abnormal expression of m^6^A effector proteins in peripheral blood mononuclear cells or biopsy specimen of DM and its microvascular complications, limited researches have not been able to confirm whether effector proteins are specifically expressed only in a particular disease or at a certain stage in the process of diseases. Therefore, they cannot be employed as a specific biomarker for early diagnosis in DM and its microvascular complications.

## 6 Conclusion and perspectives

m^6^A is a dynamic and reversible epigenetic modification. A growing number of studies have revealed m^6^A is involved in the occurrence and development of various metabolic diseases, such as obesity, cardiovascular diseases, diabetes, NAFLD and et al., and provides valuable insights into the etiology, pathogenic mechanism and treatment (168). This review summarizes the underlying molecular mechanisms between m^6^A in DM and its microvascular complications, but many mechanisms remain to be elucidated. Current research exists some limitations. First, in most studies, animal and cell models are used for *in vivo* and *in vitro* studies, while patients sample is rarely used. Second, some researchers have discovered the association of genetic variants with DM using GWAS, but have not explored the mechanism in depth. Third, dysfunction of m^6^A effector proteins have been identified in diabetic microvascular complications, however upstream regulators remain unclear. Fourth, sample size is relatively small. Of note, DKD, DR, DPN are all microvascular complications of diabetes. There are differences in the regulation of RNA methylation in different organs due to heterogeneity in terms of tissue distribution, origin, phenotype and microenvironment, which also increases the difficulty in the study between m^6^A methylation and microvascular complications of diabetes. We put forward several future research directions. First, with the advancement of gene sequencing technology and the reduction of cost, more metabolism-related genomics will be discovered so as to further explore the biological function of m^6^A and the mechanism in diabetes and its complications. Second, multiple enzymes together regulate and maintain m^6^A RNA methylation. Future studies should focus on the interaction between multiple enzymes under multiple incentive conditions and the mutual interplays of m^6^A and other RNA modifications in DM and diabetic microvascular complications. Third, exploring m^6^A modification as a specific biomarker that can predict the development of diabetes, the risk of complications, and the response to treatment will be conducive to more precise disease management and intervention. Fourth, due to the heterogeneity of diabetes, it is essential to investigate individual differences of m^6^A in patients with diabetes and develop individualized diagnosis and treatment strategies. Fifth, develop drugs that target specific pathologic pathways based on in-depth understanding of m^6^A and DM and its microvascular complications, for instance β cell protection and regeneration, podocyte repair and etc. In conclusion, discovering underlying mechanisms of m^6^A methylation in DM and its microvascular complications and more upstream regulators and downstream targets of m^6^A are beneficial for providing more personalized, effective and safe treatment strategies for diabetes patients.

## Glossary

m^6^A: N6-methyladenosine
DM: diabetes mellitus
IR: insulin resistance
DKD: diabetic kidney disease
DR: diabetic retinopathy
DPN: diabetic peripheral neuropathy
MTC: methyltransferase complex
WTAP: Wilms' tumor 1-associated protein
ZCCHC4: Zinc finger CCHC-type containing 4
RBM15: RNA-binding motif protein 15
XIST: X-inactive specific transcript
FTO: fat mass and obesity-associated protein
ALKBH5: AlkB homolog 5
YTH: YT521-B homology
HNRNPs: heterogeneous nuclear ribonucleoproteins
IGF2BPs: insulin-like growth factor 2 mRNA-binding proteins
YTHDF: YTH domain family protein
YTHDC: YTH domain containing protein
RRM: RNA recognition motif
MeRIP-Seq: methylated RNA immunoprecipitation sequencing
ncRNA: non-coding RNAs
T2D: type 2 diabetes
HFD: high fat diet
MG: methylglyoxal
GSIS: glucose-stimulated insulin secretion
MafA: musculoaponeurotic fibrosarcoma oncogene family A
OAS: 2′-5′-oligoadenylate synthetase
NAFLD: nonalcoholic fatty liver disease
DN: diabetic nephropathy
GRINCH: glucose-responsive insulin-secreting C-peptide modified human proinsulin
ROS: reactive oxygen species
EMT: epithelial-to-mesenchymal transition
GWAS: genome-wide association studies
miRNAs: microRNAs
lncRNAs: long noncoding RNAs
circRNAs: circular RNAs
EMT: epithelial-to-mesenchymal transition
ERS: endoplasmic reticulum stress
HG: high glucose
NSD2: nuclear receptor-binding SET domain protein 2
TFA: total flavones of Abelmoschus manihot
MSCs: mesenchymal stem cells
CAMK: calcium/calmodulin-dependent protein kinases
RPE: Retinal pigment epithelium
BRB: blood-retinal barrier
HRMECs: human retinal microvascular endothelial cells
rMCs: retinal Müller cells
PARP: poly (ADP-ribose) polymerase
HAGLR: HOXD Cluster Antisense RNA 1
DFU: diabetic foot ulcers
ADSCs: adipose derived stem cells.
